# Calcarea carbonica induces apoptosis in cancer cells in p53-dependent manner *via* an immuno-modulatory circuit

**DOI:** 10.1186/1472-6882-13-230

**Published:** 2013-09-21

**Authors:** Shilpi Saha, Dewan Md Sakib Hossain, Shravanti Mukherjee, Suchismita Mohanty, Minakshi Mazumdar, Sanhita Mukherjee, Uttam K Ghosh, Chaturbhuj Nayek, Chinta Raveendar, Anil Khurana, Rathin Chakrabarty, Gaurisankar Sa, Tanya Das

**Affiliations:** 1 Division of Molecular Medicine, Bose Institute, P1/12, CIT Scheme VIIM, Kolkata 700054, India; 2 Department of Physiology, Calcutta National Medical College, Kolkata 700014, India; 3 Central Council for Research in Homeopathy, 61-65, Institutional Area, Janakpuri, New Delhi 110058, India; 4 Bholanath Chakrabarty Trust, 5, Subol Koley lane, Howrah 711101, India

**Keywords:** Apoptosis, Cancer, Calcarea carbonica, Breast cancer, p53

## Abstract

**Background:**

Complementary medicines, including homeopathy, are used by many patients with cancer, usually alongside with conventional treatment. However, the molecular mechanisms underneath the anti-cancer effect, if any, of these medicines have still remained unexplored. To this end we attempted to evaluate the efficacy of calcarea carbonica, a homeopathic medicine, as an anti-cancer agent and to delineate the detail molecular mechanism(s) underlying calcerea carbonica-induced tumor regression.

**Methods:**

To investigate and delineate the underlying mechanisms of calcarea carbonica-induced tumor regression, Trypan blue dye-exclusion test, flow cytometric, Western blot and reverse transcriptase-PCR techniques were employed. Further, siRNA transfections and inhibitor studies were used to validate the involvement of p53 pathway in calcarea carbonica-induced apoptosis in cancer cells.

**Results:**

Interestingly, although calcarea carbonica administration to Ehrlich’s ascites carcinoma (EAC)- and Sarcoma-180 (S-180)-bearing Swiss albino mice resulted in 30-35% tumor cell apoptosis, it failed to induce any significant cell death in *ex vivo* conditions. These results prompted us to examine whether calcarea carbonica employs the immuno-modulatory circuit in asserting its anti-tumor effects. Calcarea carbonica prevented tumor-induced loss of effector T cell repertoire, reversed type-2 cytokine bias and attenuated tumor-induced inhibition of T cell proliferation in tumor-bearing host. To confirm the role of immune system in calcarea carbonica-induced cancer cell death, a battery of cancer cells were co-cultured with calcarea carbonica-primed T cells. Our results indicated a "two-step" mechanism of the induction of apoptosis in tumor cells by calcarea carbonica i.e., (1) activation of the immune system of the host; and (2) induction of cancer cell apoptosis *via* immuno-modulatory circuit in p53-dependent manner by down-regulating Bcl-2:Bax ratio. Bax up-regulation resulted in mitochondrial transmembrane potential loss and cytochrome c release followed by activation of caspase cascade. Knocking out of p53 by RNA-interference inhibited calcarea carbonica-induced apoptosis thereby confirming the contribution of p53.

**Conclusion:**

These observations delineate the significance of immuno-modulatory circuit during calcarea carbonica-mediated tumor apoptosis. The molecular mechanism identified may serve as a platform for involving calcarea carbonica into immunotherapeutic strategies for effective tumor regression.

## Background

Despite significant advances toward targeted therapy and screening techniques, breast cancer continues to be the leading cause of cancer-related deaths and the most frequently diagnosed cancer among women worldwide [1]. Although chemotherapy plays an important role in the treatment of breast cancer, the high percentage of failures after initial responses and the adverse toxic side effects [2] of chemotherapeutic drugs highlight the necessity of the identification of novel agents that can suppress growth of human breast cancers and are still relatively safe. In this regard, the use of complementary and alternative medicine (CAM) including homeopathic remedies is on the rise worldwide, and patients with cancer are increasingly opting to be treated with CAM therapeutic regimens [3-5]. A few reports describe the anti-cancer effect of homeopathic remedies and on their mechanism of action in experimental cancers and cell cultures [6-10]. Among conventionally used homeopathic medicine, calcarea carbonica, which is derived from the soft white middle layer of the oyster shell that is composed of fine crystalline calcium carbonate (CaCO_3_) with traces of other minerals, such as magnesium carbonate, has been reported to have *in vitro* and *in vivo* anti-cancer properties in a murine melanoma model [11]. However, the detail mechanistic studies affirming the anti-cancer effect of calcarea carbonica are still inadequate.

It is now acknowledged that the multifaceted defect in the immune capacity of patients with advanced malignancy contributes not only to disease progression but also constitutes a barrier to therapeutic interventions. Both human patients and experimental animals with advanced cancer often exhibit a poorly functioning immune system [12-15], manifested by decreased T cell proliferation [16], alteration in signal-transducing molecules [17,18], reduced CD4^+^:CD8^+^ ratios, and deficient production of Th-1 cytokines [16,19,20]. These alterations correlate with the severity of disease and with poor survival. On the other hand, activation of tumor-suppressed immune system has been observed to regress tumor *via* immuno-modulatory circuit. For example Das *et al.,* have demonstrated that soluble immune mediators like TNF-α and NO (Nitric oxide) released from spleenic cells resulted in tumor apoptosis. Importantly, many of the cancer drugs in use suppress immune system [21] thereby adding to the causes of failure of cancer therapeutic regimens. A few reports have shown that calcarea carbonica, on the other hand, possessed immuno-potentiating effects [11,22] and improved the immune response against tumor cells or even induce direct dormancy in malignancies [11]. All these information raise a possibility that calcarea carbonica may regress cancer by correcting the suppressed immune system of the tumor-bearer.

Multiple pathways have been proposed by which immune system can be stimulated to recognize and trigger cancer cell apoptosis. Cytotoxic T lymphocytes (CTL) are antigen-specific effector cells of the immune system with the ability to lyse target cells in a contact-dependent manner. Most CTL expressing antigen specific receptors (TCRs) mediate the elimination of tumor cells by recognition of antigen in the form of individual peptides bound to MHC molecules [23,24]. Operationally, apoptosis is initiated by "death receptors" (TNF receptor, Fas, DR3, DR4, and DR5), by p53-dependent and -independent cellular stress pathways that induce permeability transition in mitochondria and release of cytochrome c, and by the secretion of granules that contain perforin and granzymes from CTLs [25-28]. Studies by Dorothee *et al*. [29] suggest that lung carcinoma-specific CTLs use mainly a granule exocytosis-dependent pathway to lyse autologous target cells and that these effectors are able to circumvent alteration of the Fas-triggered intracellular signalling pathway *via* activation of a caspase-independent cytoplasmic death mechanism. Similarly Kawasaki *et al*. [30] have helped to understand the intracellular trafficking events during the very early stages of target cell apoptosis induced by CTLs. The report that calcarea carbonica improves the immune response against tumor cells [11] tempted us to hypothesize that calcarea carbonica regresses tumor *via* immuno-modulatory circuit, the molecular basis of which needs to be explored for future translational research.

In the present study we delineated the detail molecular mechanisms underlying the anti-cancer effect of calcarea carbonica. Interestingly our results indicate that although calcarea carbonica (6C) resulted in 30-35% tumor cell apoptosis when administered to Ehrlich’s ascites carcinoma (EAC) and S-180 bearing Swiss albino mice, it failed to induce any significant cell death in *ex vivo* conditions. Importantly, since calcarea carbonica 6C lessened tumor burden significantly while 12C, 30C and 200C failed to impart any decrease in tumor cell number, further studies were performed using this dose of calcarea carbonica. Moreover, while in tumor-bearing mice, there was profound depletion of CD4^+^ and CD8^+^ cells in peripheral circulation, dominance of T helper cell type-2 (Th2) that dampened T cytotoxic cell type-1 immune responses, and inhibition of T cell proliferation, calcarea carbonica protected the immune system from such tumor-insult. These results tempted us to hypothesize that calcarea carbonica might adopt a "two-step" mechanism of the induction of apoptosis in tumor cells, i.e., (1) activation of the immune system of the host, and (2) induction of cancer cell apoptosis *via* immuno-modulatory circuit. In an attempt to confirm the role of calcarea carbonica-activated immune system in cancer cell death, tumor cells were co-cultured with T cells from calcarea carbonica-administered tumor-bearing mice. Our results indicated that in comparison to untreated T cells, calcarea carbonica-activated T cells induced cancer cell apoptosis in p53-dependent manner by down-regulating Bcl-2/Bax ratio that finally culminated at the activation of mitochondrial death cascade. In summary, these observations for the first time delineate the molecular mechanism underlying immuno-therapeutic activity of calcarea carbonica against cancer that can be exploited in future to achieve efficient tumor regression *via* immuno-modulatory circuit.

## Methods

### (A) *in vivo* experiments

#### ***Placebo and drug details***

The placebo (potentized hydroalcoholic solution) and different strengths (1C, 6C, 12C, 30C and 200C) of calcarea carbonica were purchased from Hahnemann Publishing Co. Pvt. Ltd., authorized manufacturing house certified by GMP and ISO. The drugs procured were colorless, odorless, pre-sterilized and endotoxin free. The remedies were stored in brown coloured glass containers at room temperature, away from sunlight.

### Treatment of animals

**Figure 1:**
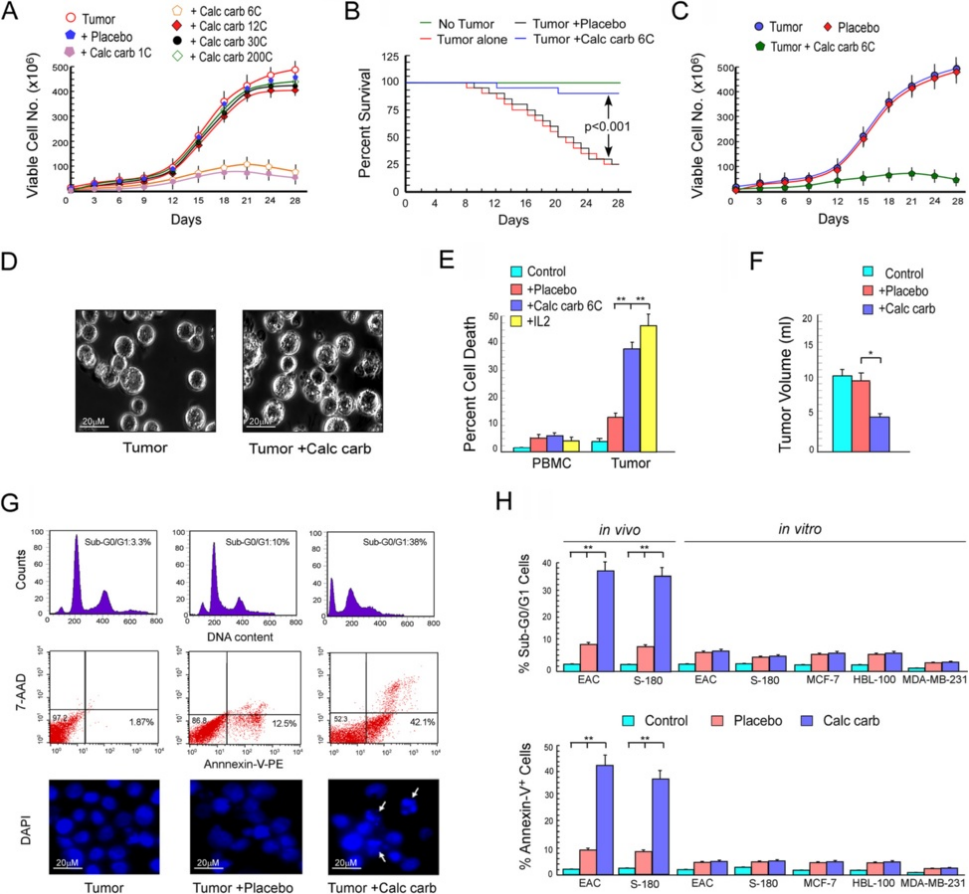
**Calcarea carbonica induced tumor apoptosis *in vivo *but not in *vitro*.** Swiss albino mice were intra-peritoneally injected with 1×10^6^ EAC (Ehrlich’s ascites carcinoma). After 1 week, placebo/calcarea carbonica (1C, 6C, 12C, 30C and 200C) were administered orally for 27 days. **(A)** Hereafter every 3 days the viable EACs were counted from the peritoneal cavity of mice and represented graphically. **(B)** Kaplan-Meir plot depicting survival rates in untreated, placebo- and calcarea carbonica-treated tumor-bearing mice. Arrow heads represent the statistical significance between survival percentages of un-/calcarea carbonica-treated tumor-bearing mice (p < 0.001). **(C)** Graphical representation of tumor cell viability after re-treatment with calcarea carbonica for 27 days to confirm that calcarea carbonica does not induce resistance. **(D)** Phase contrast images showing morphological changes of EAC cells after drug treatment. Bar length in images indicate 20 μm **(E)** At day 21 after placebo-/calcarea carbonica-/IL2-treatment percent PBMC and tumor cell death was determined by Trypan blue dye-exclusion test. **(F)** Graphical representation of tumor volume from placebo-/calcarea carbonica-treated tumor-bearing mice at day 21. **(G)** The nature of calcarea carbonica-induced tumor cell was assayed flow cytometrically using cell cycle phase distribution assay (upper panel) and Annexin-V-PE/7-AAD double labelling assay (middle panel). DAPI staining revealed nuclear morphology of apoptotic cells as indicated by arrowheads (lower panel). Bar length in images indicate 20 μm. **(H)** Graphical representation of percent apoptosis from control, untreated, placebo- and calcarea carbonica-induced murine and human cancer cell death was measured flow cytometrically under both *in vitro* and *in vivo* conditions. Values are mean ± SEM of five independent experiments. *p < 0.05 and **p < 0.001 when compared with respective control/treated groups.

Swiss albino mice (NCLAS, Hyderabad, India) weighing 20-25 g were maintained in temperature-controlled room with light–dark cycle. All animal experiments were performed following 'Principles of laboratory animal care’ (NIH publication No. 85–23, revised in 1985) as well as Indian laws on 'Protection of Animals’ under the provision of the Ethics Committee for the purpose of control and supervision of experiments on animals (Reg. No. 95/99/CPCSEA), Bose Institute. The experimental sets were as follows- 1) normal set (non-tumor bearing mice), 2) tumor-bearing set which were intra-peritoneally injected with 1×10^6^ exponentially grown p53-wild-type-Ehrlich’s ascites carcinoma (EAC), 3) placebo 6C-treated EAC-bearing set, 4) calcarea carbonica 1C-treated EAC-bearing set, 5) calcarea carbonica 6C-treated EAC-bearing set, 6) calcarea carbonica 12C-treated EAC-bearing set, 7) calcarea carbonica 30C-treated EAC-bearing set and 8) calcarea carbonica 200C-treated EAC-bearing set, 9) tumor-bearing set which were intra-peritoneally injected with 1×10^6^ exponentially grown p53-wild-type-Sarcoma-180 (S-180) and 10) placebo 6C-treated S-180-bearing set, 11) calcarea carbonica 6C-treated S-180-bearing set and 12) IL2-treated EAC-bearing set. For experiments, the results of which have been furnished in Figure 1A and 1C, each group comprised of 3 mice for each time point. For rest of the experiments, each group comprised of 5 mice.

The experiments were blind performed. Standard published protocol [11] with few modifications was followed for drug administration. To delineate the efficacy of calcarea carbonica 6C to provide survival benefit to tumor-bearing mice and to understand the refractory nature of tumors harvested from calcarea carbonica 6C-treated animals to re-treatment with the drug, calcarea carbonica was orally administered by pipetting 1 ml/kg of body-weight into the mouth of mouse, twice daily for 27 days (treatment started seven days after tumor inoculation). Besides the above mentioned experiments, for rest of the *in vivo* experiments calcarea carbonica treatment was done twice daily for 21 days. Immediately before each treatment remedies were vigorously shaken (succussed) by manually tapping on palm ten times. Care was taken to give similar pressure at each stroke.

To delineate the efficacy of calcarea carbonica 6C to provide survival benefit to tumor-bearing mice, the mice were divided into 4 groups of 10 animals each including normal set (non-tumor-bearing), tumor-bearing set (which were intra-peritoneally injected with 1 × 10^6^ exponentially grown p53-wild-type-Ehrlich’s ascites carcinoma (EAC), placebo 6C-treated tumor-bearing set and calcarea carbonica 6C-treated tumor-bearing set.

The end-point of the mice in the experiments was decided by measuring the tumor burden. The experiment was ended on day 28 of the tumor inoculation when the tumor burden was less than one-fifth of the original body-weight of mice. IL2 (300 IU/kg body-weight) was administered to tumor-bearing mice for 21 days as positive control for the anti-tumor and immune-modulating properties of calcarea carbonica 6C.

The efficacy of treatment on liquid tumors in mice was examined by measuring the changes in the peritoneal ascites volume of un-treated, placebo-/calcarea carbonica-treated tumor-bearing mice after completion of treatments.

### Transplantation and Re-treatment experiment

To transplant the tumor into new mice, 1 × 10^6^ viable Ehrlich’s ascites carcinoma (EAC) cells were inoculated into the peritoneal cavity of the mice. The EACs were sorted by negative selection using anti-CD3 and anti-CD16 antibody coated micro-beads (Milteny Biotech). Before inoculation, more than 98% of the CD16-/CD3-negative cells were morphologically characterized as EAC by Wright staining [19,20].

To understand the refractory nature of tumors to re-treatment, EACs were isolated from the peritoneal cavity of mice that had undergone 27 days of treatment with calcarea carbonica. Mice were sacrificed on the specified day and EACs were then subjected to sorting and characterization as described above. Viability was assessed by Trypan blue dye exclusion. Viable 1 × 10^6^ EAC cells were then transplanted into the peritoneal cavity of normal mice. The experimental sets included un-/placebo 6C-/calcarea carbonica 6C-treated tumor-bearing mice.

### Peripheral blood mononuclear cells (PBMC) isolation

Peripheral blood collected from mice was centrifuged over Ficoll-Hypaque (Ammersham Pharmacia) density-gradient to obtain total lymphocytes [15-17]. T cells were purified by positive selection from total lymphocytes using micro-beads coated with mouse/human anti-CD3 antibodies (Milteny Biotech).

### Phenotypic analysis of helper and cytotoxic T cells

For the determination of helper and cytotoxic T lymphocytes, T cells from thymus, spleen, lymph node and peripheral blood from normal (non-tumor bearing) mice, control, placebo- and calcarea carbonica/IL2-treated tumor-bearing mice were isolated after 21 days of treatment, and labeled with PerCP**-**conjugated CD4, PE-conjugated CD8 antibodies (BD Bioscience). Cells were then analyzed in FACS (BD Bioscience) equipped with 488 nm argon laser light source and a 675/20-nm band pass filter for PerCP-fluorescence and 575 nm band pass filter for PE-fluorescence. Cells were properly acquired, gated and analyzed using CellQuest Software (BD Bioscience). To purify CD4^+^ and CD8^+^ T cells for co-culture experiments, total T cell population isolated from normal human blood was stained with anti-CD4-PerCP and anti-CD8-PE antibodies. Stained cells were then subjected to high speed cell sorting (FACS-Aria; BD Bioscience) under sterile condition to obtain CD4^+^-depleted T cells and CD8^+^-depleted T-cell populations.

For the determination of apoptosis, total T cell population isolated after 21 days of treatment, from the peritoneal cavity of un-/placebo-/calcarea carbonica-treated mice were divided into two equal parts, one part was labeled with PerCP-CD4 and Annexin-V-FITC and propidium iodide, the other part was labeled with PE-CD8, 7-amino-actinomycin D (7-AAD) and Annexin-V-FITC (BD Bioscience) and analyzed on flowcytometer. Annexin-V^+^ cells were regarded as apoptotic cells [19,20]. To prevent the overlapping of fluorescent emission spectra of 7-AAD - PE and PerCP - PI, the spectral patterns of respective fluorochrome pairs were compensated during acquisition of flowcytometric data.

### Proliferation assay

The CD3^+^ cells isolated from peripheral blood of normal mice (non-tumor bearing), control (un-treated), placebo treated- and calcarea carbonica-treated tumor bearing mice after 21 days of treatment, were loaded with 5-(and-6)-carbonicaoxy fluorescein succinimidyl ester (CFSE; Molecular Probe) and proliferation was assessed by stimulating CD3^+^ cells (1 × 10^6^ cells/ml) in combination with cross-linked anti-CD3 antibody and soluble anti-CD28 antibody for 72 h. Decrease in CFSE-fluorescence as marker of cell proliferation was assayed flow cytometrically [16].

### Cell cycle phase distribution and apoptosis assay

For the determination of cell cycle phase distribution of DNA content, EAC cells harvested from the peritoneal cavity of un-/placebo-/calcarea carbonica-treated mice tumor-bearing mice were permeabilized and nuclear DNA was labelled with propidium iodide (PI) using Cycle TEST PLUS DNA reagent kit. Cell cycle phase distribution of nuclear DNA was determined on FACS, fluorescence detector equipped with 488 nm argon laser light source and 623 nm band pass filter (linear scale) using CellQuest software (Becton Dickinson). A total of 10, 000 events was acquired and analysis of flowcytometric data was performed using ModFit software. A histogram of DNA content (x-axis, PI fluorescence) versus counts (y-axis) has been displayed [21].

For DAPI staining cell were fixed in 3% *p*-formaldehyde/Triton-×100 and stained with 4’,6-diamidino-2-phenylindole (DAPI; Pharmingen). A Leica fluorescent microscope DM 900 was used to visualize the fluorescent images. Digital images were captured with a highly sensitive cool (-25°C) charged coupled device camera (Princeton Instruments) controlled with the MetaMorph software (Universal Imaging).

### Flow cytometric detection of intracellular cytokine

T cells isolated from peripheral blood, spleen, lymph node and thymus of non-tumor bearing normal mice, control (un-treated) and placebo-/calcarea carbonica-treated tumor bearing mice after 21 days of placebo-/drug-treatment were stimulated with phorbol-12-myristate-13-acetate (PMA; 10 ng/ml) and ionomycin (1 μM) (Sigma). After incubation for 4 h at 37°C cells were washed with PBS and half of the cells were labeled with PerCP-CD4 or PerCP-CD8 antibodies. Cells were permeabilized with saponin and intracellular IFN-γ, and IL-4 (10 μl, dilution 1:30; BD Bioscience) were labeled with PE-/FITC-tagged antibodies and were analyzed in FACS. Type-2 bias is defined as the ratio of cells producing type-2 cytokine (IL-4) divided by the proportion of cells producing type-1 cytokine (IFNγ) [16].

### (B) *in vitro* experiments

#### ***Cell culture***

p53-wild-type-MCF-7, -HBL-100 and p53-mutated-MDA-MB-231, human breast cancer cells were obtained from NCCS and routinely maintained in complete RPMI 1640 medium at 37°C in humidified incubator containing 5% CO_2_[31,32]. Furthermore, to determine the role of p53 in calcarea carbonica-induced apoptosis, EAC-p53-deficient cells and p53-silenced MCF-7 cells were utilized. The p53-silencing was done by transfecting EAC cells with p53-shRNA (small hairpin) and permanent clones (EAC-p53-deficient cells) were selected by culturing the transfectants with G418 (400 μg/ml) for 2 weeks with passaging after each 3rd day [33,34]. The p53-deficient-EAC cells were maintained in 200 μg/ml of G418 and then injected in the peritoneal cavity of mice. Transient p53-silencing was done by transfecting MCF-7 cells with p53-siRNA (small interfering) following manufacturer’s instructions. The p53-siRNA/shRNA transfection efficacy in EAC/MCF-7 cells was validated by Western blot analysis.

### Co-culture experiments

For co-culture experiments isogenic conditions were maintained, i.e., T cells isolated from peripheral blood of mice were co-incubated with cancer cells of mice origin, EAC and S-180 cells and human peripheral T cells were co-cultured with breast cancer cells of human origin (MCF-7, HBL-100, MDA-MB-231). Prior to incubation with target cells, T cells isolated from normal human donors were cultured in anti-CD3/anti-CD28 coated culture plates in media alone (control), tumor spent medium (un-primed) placebo-treated-(placebo-primed) and calcarea carbonica-treated-tumor spent medium (calcarea carbonica-primed). For priming T cells, tumor spent medium were treated with 20 μl/ml of placebo-/calcarea carbonica 6C. Tumor spent medium is 72-hour old cell-free tumor supernatants used for co-culture experiments to mimic the tumor-bearing condition in which tumor shed mediators influence the circulating T cell repertoire. After 3 days these control, un-/placebo-/calcarea carbonica-primed T cells were co-cultured with breast cancer cells (MCF-7, HBL-100, MDA-MB-231) for 48 hrs.

To examine the effect of varying effector-to-target ratio, CD3^+^ T cells isolated from peripheral blood of control or placebo-/drug-treated mice were incubated with EAC cells for 48 hrs at effector-to-target ratio of 5:1, 10:1 and 50:1 and percent apoptosis was scored by Annexin-V/7-AAD assay. In another experiment, human T cells and T cell-free supernatants after priming were co-incubated with MCF-7 cells to understand the requirement of T cell-tumor cell contact during T cell-mediated tumor killing. After 48 hrs, MCF-7 cells from both the sets were scored for percent apoptosis employing Annexin-V/7-AAD assay.

### Treatment of cells

During all *in vitro* experiments, cancer cells were treated with 20 μl/ml calcarea carbonica 6C. To understand the sequence of events leading to apoptosis, cancer cells were treated with mitochondrial pore inhibitor CsA (25 μM; Merck, Germany) for 1 h prior to calcarea carbonica treatment and with 50 μM of caspase-3 inhibitor Z-DEVD-FMK and caspase-9 inhibitor Z-LEHD-FMK (Calbiochem), 3 h prior to incubation with calcarea carbonica.

### Flow cytometric measurement of mitochondrial membrane potential

For measurement of mitochondrial transmembrane potential (MTP) loss, cells were loaded with potential-sensitive dye Dihexyloxacarbonicao cyanine (DiOC_6_, Merck, Germany) during the last 30 min of treatment at 37°C in the dark. Fluorescence of retained DiOC_6_ was determined flow cytometrically using logarithmic amplification by CellQuest software (Becton Dickinson).

### Western blot

Cell lysates were prepared in lysis buffer [20 mMTris–HCl (pH 7.4), 100 mM NaCl, 1% NP40, 0.5% sodium deoxycholate, and 1 mM EGTA] containing protease inhibitors. Mitochondrial and cytosolic fractions were prepared according to Yamaguchi and Wang [35]. A total of 50 μg of protein was separated by SDS-PAGE and transferred to nitrocellulose filter paper for Western blotting using specific antibodies e.g., anti-p53 (DO-1), anti-Bcl-2 (N-19), anti-Bax (N-20), anti-cytochrome c (C-20), caspase-3 (E-8), caspase-9, anti-MnSOD (N-20) from Santa Cruz. The blots were developed by chemiluminescence (1:1) [33,34]. In parallel experiment equivalent amount of protein was Western blotted with anti-α-actin antibody (C-2; Santa Cruz) to confirm equal protein leading.

### siRNA, transfections and RT-PCR

Cells were transfected with 300pmole of p53-/caspase-3-/control-ds-siRNA or p53-shRNA (Santa Cruz) and lipofectamine-2000 separately for 12 h. The protein levels of p53-/caspase-3-were estimated by Western blotting. For RT-PCR assay, 2 μg of total RNA, extracted with TRIzol reagent, was reverse-transcribed and then subjected to PCR with enzymes and reagents of the RTplusPCR system (Eppendorf, Hamburg, Germany) using GeneAmp PCR system 2720 (Applied Biosystems; Foster City) [31-34]. Primers for Bcl-2 were 5′-CTGGCATCTTCTCCTTCCAG-3′ and 5′-GACGGTAGCGGACGAG-AGAAG-3′; Bax were 5′-TTTGCTTCAGGGTTTCATCC-3′ and 5′-CAGTTGAAGTTGCCGTCAGA-3′; and GAPDH (internal standard) were 5′-CAGAACATCATCCCTGCCTCT-3′ and 5′-GCTTGACAAAGTGGTCGTTGA G-3′.

### Explant assay

#### ***Isolation and culture of primary breast cancer cells***

Normal breast tissue or primary lesions of breast cancer were obtained from patients with localized disease after prior written informed consent under the provision of Ethics committee, Calcutta National Medical College, Kolkata, India (Approval letter No: CNMC/ETHI/162/P) and Human Ethics Committee, Bose Institute (Approval letter No: BIHEC/2010-11/2). The selected cases consisted of 5 primary breast cancer patients that had not been treated with chemotherapy or radiation. Normal mammary epithelial tissue of the same patient was used as the control. The specimens were washed with phosphate buffered saline, cut into small pieces, 5×5 mm in size, and immersed in a mixture of colloagenase (10%, Calbiochem) and hyaluronidase (0.5 mg/ml, Calbiochem) for 12-16 h at 37°C on orbital shaker. The contents were then centrifuged at 80×g for 30 sec at room temperature. The supernatant comprising mammary fibroblasts were discarded and in the pellet pre-warmed 0.125% trypsin-EDTA was added. The mixture was gently pipetted and kept for 30 min at 37°C. Finally the pellet obtained was washed with cold Hank’s buffer saline with 2% fetal bovine serum and centrifuged at 450×g for 5 min at room temperature. Then the single cells were seeded on poly-L lysine coated dishes and cultured in a serum-free medium containing growth factors, 0.1 ng/ml human recombinant epidermal growth factor, 5 μg/ml insulin, 0.5 μg/ml hydrocortisone, 50 μg/ml gentamycin, 50 ng/ml amphotericin-B, and 15 μg/ml bovine pituitary extract at 37°C. Medium was replaced every 4 days and passages were done when the cells reached, 80% confluence**.** Peripheral blood was obtained from healthy volunteers and patients after prior written informed consent under the provision of Ethics committee, Calcutta National Medical College, Kolkata, India (Approval letter No: CNMC/ETHI/162/P) and Human Ethics Committee, Bose Institute (Approval letter No: BIHEC/2010-11/2).

### Statistical analysis

Values are shown as standard error of mean (SEM) except otherwise indicated. Data were analyzed and, when appropriate, significance of the differences between mean values was determined by a Student’s *t* test. Results were considered significant at p <0.05.

## Results

### Calcarea carbonica inhibited tumor growth and increased survival rates of tumor-bearing mice

To identify the optimal strength of calcarea carbonica, EAC-bearing mice were administered with different strengths of drug (1C, 6C, 12C, 30C and 200C) for 27 days and anti-tumor efficacy was determined by examining any change in viable EAC cell number (Figure 1A). Non-tumor bearing untreated- and placebo 6C treated EAC-bearing mice served as control. It was witnessed that calcarea carbonica 1C and 6C lessened tumor burden significantly while 12C, 30C and 200C failed to impart any decrease in tumor cell number. Since calcarea carbonica at 1C formulation manifested problems related to solubility therefore further experiments were performed with calcarea carbonica 6C. In fact, at day 28 a total of 487 × 10^6^ tumor cells were measured in the peritoneal fluid of untreated or placebo-treated mice, whereas in calcarea carbonica-treated group tumor cell count reduced to 75 × 10^6^ (Figure 1A). Next we examined the survival benefit of calcarea carbonica to EAC-bearing mice orally fed with the same for 27 days, twice daily. The results furnished in Figure 1B depict that the tumor burden decreased the survival rates of the mice to 12.5% at day 28. Conversely, when compared to untreated or placebo-treated sets, calcarea carbonica provided a survival benefit of 88% thereby suggesting the role of calcarea carbonica administration in improving the survival rates of tumor-bearing mice (Figure 1B).

To further understand the refractory nature of tumors harvested from calcarea carbonica-treated animals to re-treatment with the drug, if any, we collected EAC cells from mice that had undergone 27 days treatment with calcarea carbonica, and transplanted them into the peritoneal cavity of other mice. After 7 days of tumor inoculation, these mice were orally administered with calcarea carbonica, twice daily for another 27 days. Change in EAC cells number was scored using trypan-blue dye exclusion assay. The findings deciphered that calcarea carbonica-treated tumors were sensitive to further treatment with this drug without development of any resistance (Figure 1C).

### Calcarea carbonica induced apoptosis in tumor cells *in vivo*

Role of calcarea carbonica in increasing the survival rate of tumor-bearing mice prompted us to explore the mechanism of its action. Our results depicted that calcarea carbonica treatment significantly depleted tumor cell number. Importantly, calcarea carbonica treatment did not show any toxicity to peripheral blood mononuclear cells (PBMCs) (Figure 1E) indicating that cytotoxic effect is specific for EAC cells. To compare cell death between PBMCs and EACs, percent cell death was calculated from the numbers of viable and dead cells in each case and represented graphically in Figure 1E. This decrease is consistent with reduced ascetic fluid volume over untreated or placebo-treated mice (Figure 1F). The decrease in EAC cell number was at equivalence with that observed in IL2-treated tumor-bearing mice (Figure 1E). IL2-fed tumor-bearing mice served as positive control to estimate the anti-tumor property of calcarea carbonica. Further morphological changes depicting cell shrinkage and blebbing in EAC cells (Figure 1D) compelled us to explore the nature of cell death. Our results revealed significant increase in the hypoploid (sub-G0/G1) DNA content of tumor cells isolated from the peritoneal cavity of EAC-bearing mice that underwent 21 days of calcarea carbonica-treatment (Figure 1G) suggesting nuclear DNA breakdown that occurs during apoptosis. Next, to confirm the nature of cell death as apoptosis, we utilized double labeling techniques using Annexin-V-PE/7-AAD to distinguish between apoptotic and necrotic cells. Our flow cytometric data revealed Annexin-V-PE-binding in EAC cells after 21 days of calcarea carbonica treatment when compared with placebo-treated EAC cells (Figure 1G) indicating that the mode of cell death is apoptosis and not necrosis. It was observed that this remedy induced apoptosis in more than 40% of tumor cells as compared to 12.5% apoptosis in placebo-treated tumor-bearing mice. In addition, tumor cells isolated from calcarea carbonica-treated EAC-bearing mice after 21 days of drug treatment when stained by DAPI, revealed a significant nuclear membrane blebbing, typical characteristic feature of apoptotic cells, when compared with tumor cells isolated from placebo-treated EAC-bearing mice (Figure 1G). These findings supported the notion that calcarea carbonica, asserts apoptogenic effect in cancer cells. Interestingly, calcarea carbonica did not furnish any significant toxic effect on peripheral circulatory immune cells of the tumor-bearing mice. Importantly, this apoptogenic effect of calcarea carbonica was not cell line-specific since S-180-bearing mice also manifested significant tumor cell apoptosis (30%) when treated with calcarea carbonica (Figure 1H).

### Calcarea carbonica failed to induce cancer cell apoptosis *ex vivo*

Our observation that calcarea carbonica induced significant apoptosis when administered *in vivo* to EAC-bearing mice model, prompted us to unveil the molecular mechanism of such anti-tumor effect of this homeopathic medicine. For the same, the effect of calcarea carbonica in different tumor cells (wild-type-p53 expressing cancer cells EAC, S-180, MCF-7, HBL-100 and p53-mutated MDA-MB-231) was determined in *in vitro* conditions by exposing tumor cells with 20 μl/ml of calcarea carbonica for a period of 96 h. Surprisingly, calcarea carbonica failed to induce cancer cell apoptosis in *ex vivo* models as evidenced by flow cytometric determination of cell cycle phase distribution and Annexin-V-PE/7-AAD double labeling assays (Figure 1H).

These results demonstrated that calcarea carbonica-mediated tumor apoptosis was restricted to *in vivo* conditions thereby indicating that the anti-cancer effect of calcarea carbonica may not be a direct one. We hypothesized that calcarea carbonica may be exploiting cell-mediated immune system to indirectly target cancer cells.

### Calcarea carbonica restored tumor-induced depletion of T cells in tumor-bearing host

**Figure 2:**
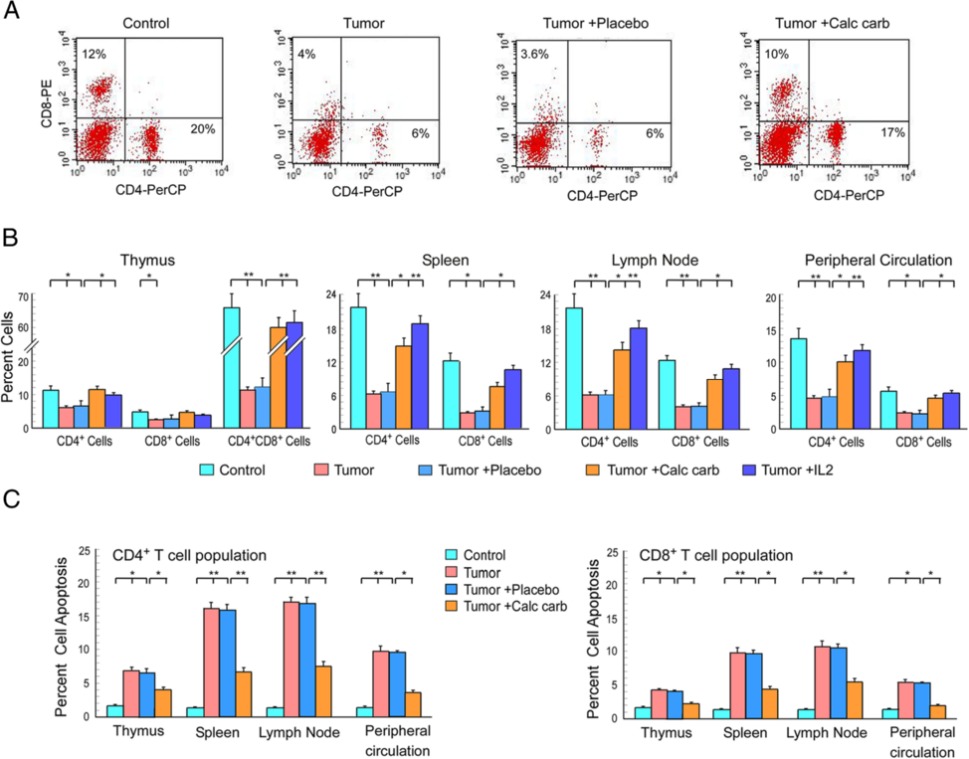
**Calcarea carbonica restores tumor-induced depletion of T cell populations.** Lymphocytes from thymus, spleen, lymph-node and peripheral circulation of untreated or placebo-/calcarea carbonica-treated tumor-bearing mice were analyzed flow cytometrically to quantitate percentages of CD4^+^/CD8^+^ populations. **(A)** Flow cytometric dot plot shows CD4^+^ and CD8^+^ T cell population in the draining lymph node of untreated or placebo-/calcarea carbonica-treated tumor-bearing mice. **(B)** Graphical representation of flow cytometric data showing percentages of CD4^+^ and CD8^+^ T cells in thymus, spleen, lymph-node and peripheral circulation of untreated or placebo-/calcarea carbonica-/IL2-treated tumor-bearing mice. **(C)** Percent CD4^+^ and CD8^+^ T-cell apoptosis (Annexin-V-positivity) from the same experimental sets were determined flow cytometrically at 21 day of drug treatment. The values are mean ± SEM of five independent experiments. *p < 0.05 and **p < 0.001 when compared with respective non-tumor/tumor-bearing control sets and placebo/drug-treated sets.

To test whether calcarea carbonica targets immune system to regress tumor burden, we next aimed to examine the effect of this drug in immune system of the tumor-bearing host. It is known that T cells play a pivotal role in cell-mediated tumor immunity, and tumors induce T cell apoptosis as a mechanism to evade the host defense system [12-15]. Thymus is the major organ where the T cell maturation process takes place, whereas spleen and draining lymph nodes are the secondary sites where antigen presentation process occurs and subsequently mature effector T cell repertoire enter the peripheral circulation. Closer scrutiny of thymus, spleen and lymph node revealed decreased percentage of CD4^+^CD8^+^ double positive as well as CD4^+^ or CD8^+^ single positive effector populations in tumor-bearing animals (Figure 2A &2B). Loss of the CD4^+^CD8^+^ double positive populations may cause a decrease in the number of mature effector cells emerging from the thymus while loss of single positive cells may result in decrease of potent T cells evolving from lymph node and spleen. Supporting this notion, CD4^+^ or CD8^+^ circulatory effector T cell populations were also severely decreased in peripheral blood of the tumor-bearing mice (Figure 2B). These results indicate that tumor-induced T cell depletion encompasses primary, secondary and effector immune compartments of the host, as decreased thymic, spleenic and lymph node output results in disruption of the circulating T cell repertoire. Interestingly, 21 days of calcarea carbonica-treatment ameliorated CD4^+^-/CD8^+^-positive effector T cell populations of thymus, spleen, lymph node as well as circulatory effector T cell populations to normal level (Figure 2A &2B). To estimate the immune-modulating effect of calcarea carbonica, we included know immunostimulatory cytokine IL2-treated tumor-bearing mice in the study. Overall our results indicate that calcarea carbonica protected the effector T lymphocytes in primary and secondary immune compartments, thereby normalizing the pool of peripheral T cell repertoire in tumor-bearer. Interestingly, calcarea carbonica showed almost similar effect when compared with IL2 (Figure 2B).

The massive depletion of T cell populations in tumor condition prompted us to investigate the underlying cause. Our flow cytometry data revealed a significant increase in Annexin-V-FITC/PI-positive CD4^+^ T-helper and Annexin-V-FITC/7-AAD-positive CD8^+^ T cytotoxic cells isolated from all the compartments of EAC-bearing mice (Figure 2C), which indicates apoptotic cell death as one of the major causes behind tumor-induced depletion of T cell repertoire. Interestingly, calcarea carbonica administration prevented apoptosis of both CD4^+^ and CD8^+^ T cells (Figure 2C). All these results suggested that calcarea carbonica has immunoprotective ability during carcinogenesis.

### Calcarea carbonica normalized Th1/Tc1-type cytokine-producing T-effector cell populations in tumor-bearing host

**Figure 3:**
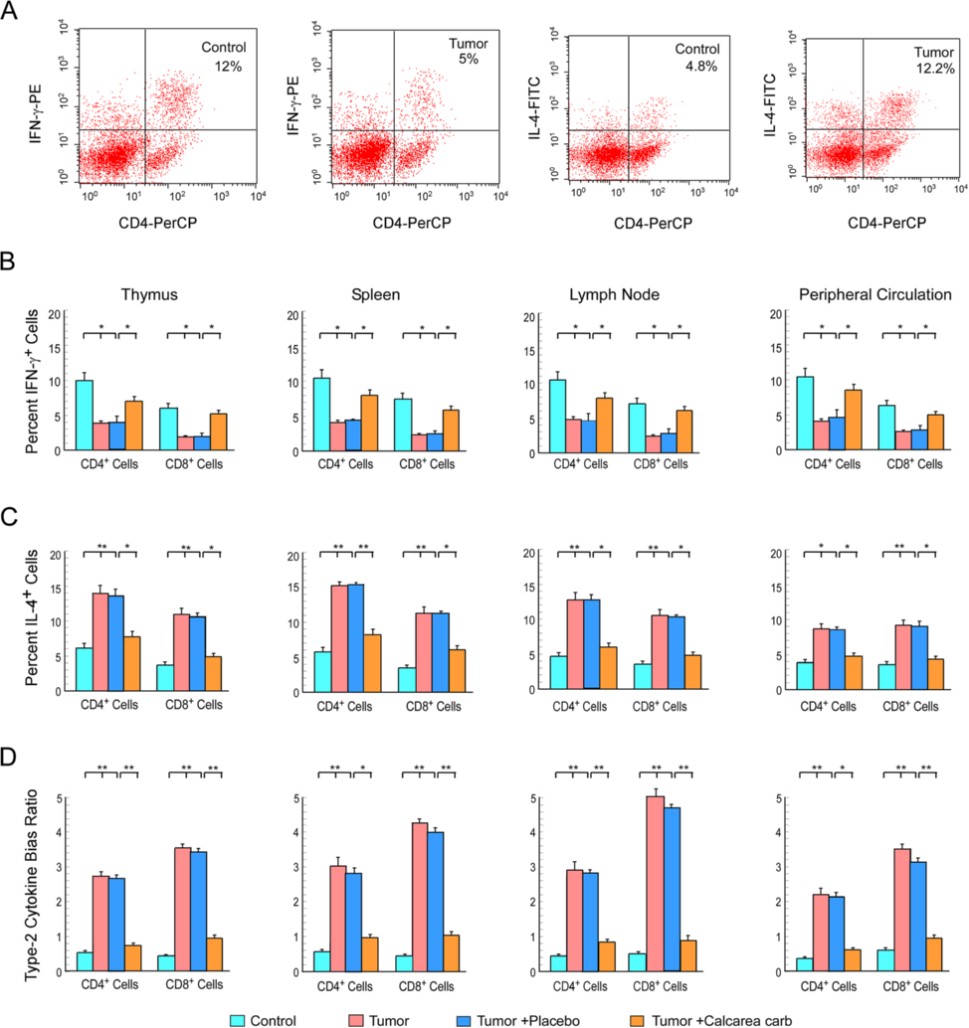
**Calcarea carbonica normalizes cytokine-producing effector T cell populations in tumor-bearing host. (A)** Typical flow cytometric patterns for IFN-γ and IL-4 producing CD4^+^ T cells in draining lymph nodes of mice. Graphical representation of flow cytometric data of intracellular IFN-γ **(B)**, IL-4 **(C)** and type-2 cytokine bias ratio **(D)** in CD4^+^/CD8^+^ T cells of thymus, spleen, lymph node and peripheral circulation of untreated or placebo-/calcarea carbonica-treated tumor-bearing and normal mice at 21 days of drug administration. Values are mean ±SEM of five independent experiments. *p < 0.05 and **p < 0.001 when compared with respective non-tumor/tumor bearing control sets and tumor bearing placebo/drug-treated sets.

It is known that Th1 and Th2 play important role in regulating cell-mediated immune system among which Th1 pathways typically produce activation of cytotoxic T lymphocytes (Tc) *via* helper T cells (Th). Cytotoxic T lymphocytes attack cancer cells to defend against tumors while Th2-mediated immunity favors tumor growth, both by promoting angiogenesis and by inhibiting cell-mediated immunity [36]. The capacity of the CD4^+^ lymphocytes isolated from lymph-node of normal and EAC-bearing mice to produce cytokines after PMA/ionomycin activation was analyzed (Figure 3A). We observed reduced proportion of Th1/Tc1-type cytokine (IFN-γ) secreting CD4^+^/CD8^+^ T cell (Figure 3B) and increased proportion of Th2/Tc2-type cytokine (IL-4) secreting CD4^+^/CD8^+^ T cell (Figure 3C) populations amongst the thymus, lymph node, spleen and peripheral circulation of EAC-bearing mice in comparison to that from their normal counterparts. Administration of calcarea carbonica to the tumor-bearers for 21 days however restored such alterations of T cells in all the compartments (Figure 3B &3C). Therefore, establishment of type-2 cytokine bias in primary and secondary immune compartments of tumor-bearing host could be efficiently reversed by calcarea carbonica (Figure 3D).

### Calcarea carbonica prevented down-regulation of T cell proliferation in tumor condition

**Figure 4:**
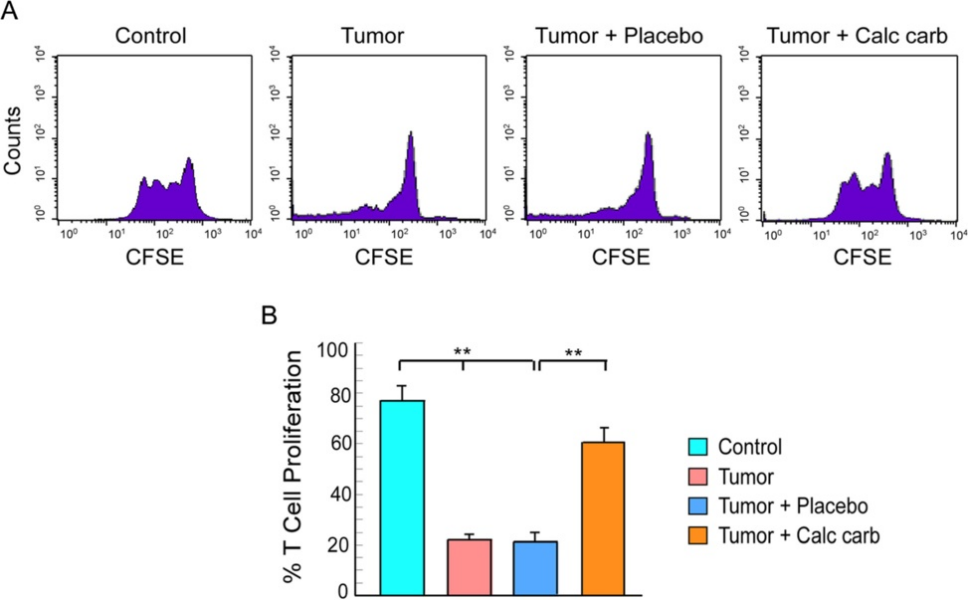
**Calcarea carbonica attenuates tumor-induced inhibition of T cell proliferation.** Flow cytometric histogram display of CFSE-fluorescence dilution **(A)** and its graphical representation **(B)** to determine CD3/CD28-stimulated effector T cell proliferation from untreated and placebo-/calcarea carbonica-treated tumor-bearing mice at 21 days of drug administration. Values are mean ±SEM of five independent experiments. **p < 0.001 when compared with respective non-tumor/tumor-bearing control sets and placebo/drug-treated sets.

Our results till now demonstrated the loss of effector T cells and increase in type-2 cytokine bias in tumor-bearing animals, which was successfully reverted back to its normal levels on calcarea carbonica administration to tumor-bearing mice. Optimal T-cell mediated anti-tumor activity likely occurs through proliferation and expansion of effector T cells. Thus, to verify if tumor also influenced the proliferative capacity of effector T cells to TCR stimulus, CD3^+^ peripheral T cells from normal/tumor-bearing/placebo-/calcarea carbonica-fed mice were loaded with CFSE for various periods of time. A CFSE division profile of anti-CD3/anti-CD28 antibody-stimulated T cells isolated from control and tumor-bearing mice is shown in Figure 4A. Discrete division cycles could be visualized by means of different CFSE signal peaks in case of T cells isolated from healthy mice, whereas T cells from tumor-bearing mice failed to proliferate in response to anti-CD3/anti-CD28 antibody. Remarkably, 21 days of calcarea carbonica-treatment prevented tumor-induced inhibition of T cell proliferation (Figure 4A &4B), which suggests that apart from preventing tumor-induced loss of effector T cells, calcarea carbonica also restored T cell proliferative capacity.

### Calcarea carbonica potentiated T cell-mediated tumor cell killing *in vitro*

**Figure 5:**
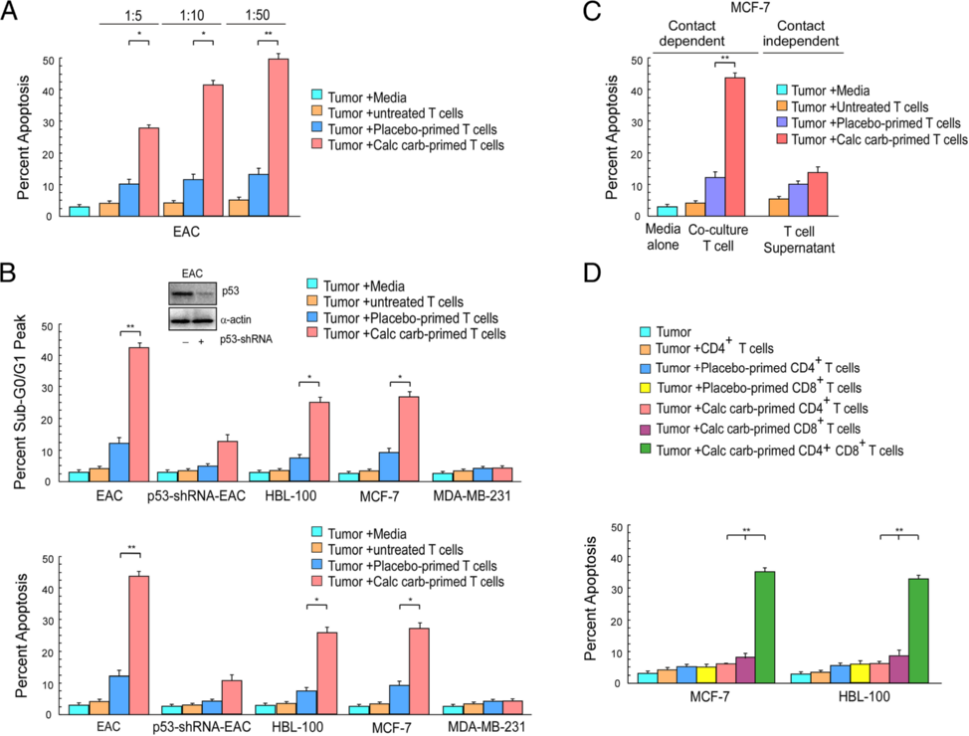
**Calcarea carbonica potentiates T cell-mediated cancer cell killing *in vitro*. (A)** Percent apoptosis of EAC cells co-cultured with T cells at multiple effector to target ratios (5:1, 10:1 and 50:1), isolated from untreated or placebo-/calcarea carbonica-treated tumor-bearing mice. Further for all co-culture experiments effector to target ratio was kept as 10:1. **(B)** Graphical representation of sub-G0/G1 populations and Annexin-V-positive populations amongst EAC and p53-silenced EACs co-cultured with or murine T cells from untreated or placebo-/calcarea carbonica-treated tumor-bearing mice. Similarly human breast cancer cell lines, MCF-7/HBL-100/MDA-MB-231 cells were co-cultured with human T cells for 48 h to determine tumor cell apoptosis. Prior to co-culture, these human T cells were primed with placebo-/calcarea carbonica-treated tumor supernatant for 72 h. The transfection efficacy of p53-shRNA was determined by Western blot (inset). **(C)** MCF-7 cell apoptosis when co-incubated with placebo-/calcarea carbonica-primed human T cells (contact-dependent) or with supernatants of placebo-/calcarea carbonica primed T cells (contact-independent) was determined flow cytometrically. **(D)** Percent apoptosis of MCF-7/HBL-100 cells when co-cultured with placebo-/calcarea carbonica-primed CD4^+^ and/or CD8^+^ T cells. Values are mean ±SEM of five independent experiments. *p < 0.05 and **p < 0.001 when compared with respective tumor-bearing control/drug-treated sets and placebo-treated/drug-treated sets.

In the light of the results obtained so far, we further validated the hypothesis that calcarea carbonica induces tumor killing *via* immuno-modulatory circuit. For the same we mimicked *in vivo* conditions by isolating peripheral CD3^+^ T cells from control, un-/placebo- and calcarea carbonica-treated EAC-bearing mice after completion of 21 days treatment and co-culturing them with EAC cells in *in vitro* conditions, at an effector to target ratio of 5:1, 10:1 and 50:1 for 48 hrs. It was observed that percent apoptosis of target tumor cells as scored by Annexin-V-PE/7-AAD double labeling assay was directly proportional to the number of effector cells (Figure 5A). We performed further co-culture experiments with 10:1 effector to target ratio as it was feasible and efficient to induce cell death in target tumor cells. Our flow cytometry data revealed that T cells from un-treated and placebo-treated EAC-bearing animals were inefficient mediators of tumor cell killing while significant increase in numbers of hypoploid and Annexin-V-positive cancer cells, co-cultured with CD3^+^ T cells isolated from calcarea carbonica-treated EAC-bearing mice, were observed (Figure 5B). Interestingly CD3^+^ T cells isolated from calcarea carbonica-treated EAC-bearing mice when co-cultured with EAC-p53-shRNA cells (p53-deficient-EAC) for 48 hrs, failed to induce apoptosis, signifying p53-dependent cell killing. To further reinstate the above hypothesis we evaluated the anti-tumor effects of control (media-alone) and tumor-supernatant pre-exposed human anti-CD3/CD28-stimulated T cells upon placebo-/calcarea carbonica-treatment for 72 hrs. Our results demonstrate that in comparison to T cells incubated with untreated and placebo-treated tumor supernatant, T cells primed with calcarea carbonica-treated tumor supernatant were efficient inducers of apoptosis in wild-type p53-expressing human breast cancer cells like MCF-7 and HBL-100 (Figure 5B) when co-cultured for 48 h. Interestingly, p53-mutated MDA-MB-231 cells resisted T cell-mediated apoptosis even in the presence of calcarea carbonica (Figure 5B). These findings further reinforced the opinion that calcarea carbonica contributes to immunocyte-mediated tumor elimination specifically in a p53-dependent manner.

To understand whether T cell-tumor cell contact is required for effective anti-tumorigenic potential of drug-primed T cells we undertook two approaches in co-culture experiments. First, to validate contact-dependent tumor killing, T cells isolated from peripheral blood of human volunteers after stimulation with anti-CD3/CD28, were subjected to four experimental sets comprising of (a) T cells cultured in media alone (control), (b) T cells cultured in untreated tumor supernatant (un-primed), (c) T cells cultured in placebo-treated tumor supernatant (placebo-primed) and (d) T cells cultured in calcarea carbonica-treated tumor supernatants (calcarea carbonica-primed) for 3 days. After 3 days, control T-cells, un-primed, placebo-primed and calcarea carbonica-primed T cells were co-cultured with breast cancer cells, MCF-7, for 48 hrs. Secondly, to examine the effect of contact-independent mechanisms during T cell-mediated tumor killing, T cells isolated from all the four experimental set were stimulated for 4 h using PMA (10 ng/ml) and ionomycin (1 μM). On the 3^rd^ day cells were centrifuged to obtain T cell-free supernatants and MCF-7 cells were co-incubated with these supernatants. After 48 hrs MCF-7 cells from both the sets were scored for percent apoptosis using Annexin-V/7-AAD assay (Figure 5C). Our findings revealed that calcarea carbonica-primed T-cells when co-cultured with breast cancer cells resulted in significant cell death, whereas calcarea carbonica-treated T cell free supernatants failed to reflect the same effect when compared with placebo data sets. Altogether these results manifest that T cell-tumor cell contact is definitely required for efficient tumor killing upon calcarea carbonica treatment.

To further delineate the therapeutic potential of T cell subsets, CD4^+^-depleted and CD8^+^-depleted T cells were utilized. To this end, human T cells isolated from peripheral blood were sorted for CD4^+^ T cells (helper T cells) and CD8^+^ T cells (cytotoxic T cells) under sterile condition. Both T cell fractions were then cultured in untreated and placebo-/calcarea carbonica-treated cell free tumor supernatants for 3 days. Tumor cells (MCF-7/HBL-100) were then co-incubated with unprimed- and placebo-/calcarea carbonica-primed CD4^+^ and CD8^+^ T cells for 48 hrs. To exclude the possibility of loss of T cells function due to the sorting procedure enriched CD4^+^ and CD8^+^ T cell subsets were mixed and incubated with tumor cells. Remarkably mixed populations showed restored anti-tumor functions. Percent apoptosis induced by CD4^+^ T_H_ cells, CD8^+^ T_C_ cells and mixed CD4^+^ and CD8^+^ sorted T cells were scored by Annexin-V/7-AAD positivity (Figure 5D). These results demonstrate that as compared to the total T cells repertoire, percent apoptosis induced by individual CD4^+^ and CD8^+^ T cells was significantly less. These observations signified the importance of both populations for efficient tumor cell killing.

### Calcarea carbonica-primed T cells induced cancer cell apoptosis in p53-dependent manner

**Figure 6:**
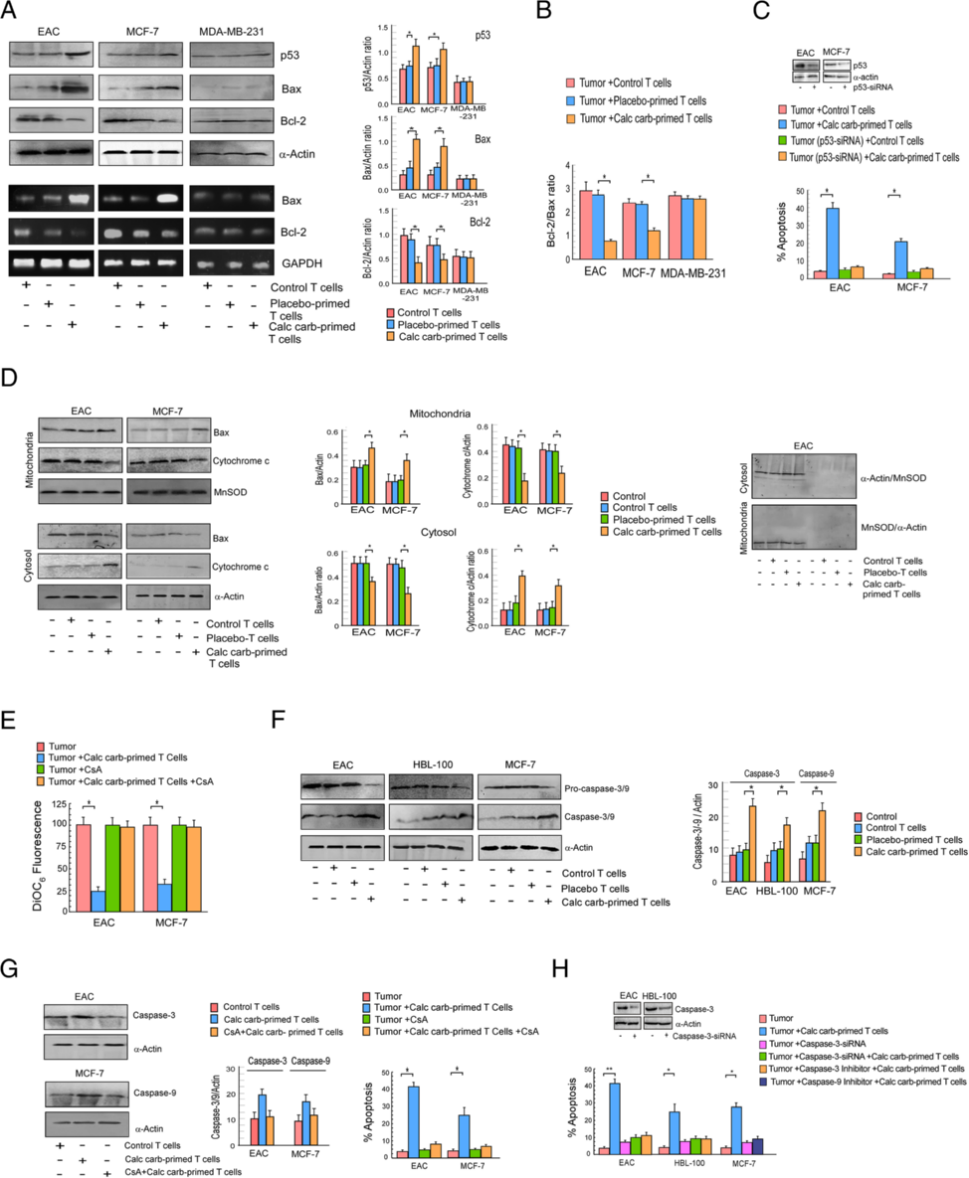
**Calcarea carbonica triggers T cell-mediated tumor killing via p53-Bax-caspase-3 cascade. (A)** EAC, MCF-7 and MDA-MB-231 cells were co-cultured with untreated-/placebo-/calcarea carbonica-primed T cells and subjected to Western blot/RT-PCR analysis to determine the expression profile of p53/Bax/Bcl-2 at protein and Bax/Bcl-2 at mRNA levels (left panels). Right panels represent quantitative data for Western blot. **(B)** Graphical representation of Bcl-2/Bax protein ratio in tumor cells co-cultured with calcarea carbonica-primed T cells. **(C)** Wild-type p53-expressing cells were transfected with p53-siRNA (inset) and scored for percent apoptosis when co-cultured with calcarea carbonica-primed T cells. **(D)** Bax and cytochrome c levels were determined in cytosolic and mitochondrial fractions of tumor cells co-cultured with placebo-/calcarea carbonica-primed T cells by Western blot analysis (left panels). Middle panels represent quantitative data. α-Actin and MnSOD were used as internal protein markers (right panels). **(E)** Graphical representation of mitochondrial trans-membrane potential of tumor cells co-cultured with calcarea carbonica-primed T cells pre-treated with cyclosporine-A. **(F)** Expression profiles of pro-/active- forms of caspase-3 in EAC and HBL-100 cells and pro-/active caspase-9 in caspase-3-null MCF-7 cells co-cultured with calcarea carbonica-primed T cells. Right panels represent quantitative data. **(G)** Expression profiles of active caspase-3 in EAC and caspase-9 in MCF-7 cells co-cultured with calcarea carbonica-primed T cells pre-treated with cyclosporine-A (left panel). Middle panel represent quantitative data. In parallel set, cells were scored for percentage apoptosis (right panel). **(H)** Percent apoptosis of EAC and HBL-100 cells co-cultured with calcarea carbonica-primed T cells in the presence of caspase-3 inhibitor (Z-DEVD-FMK) or transfected with caspase-3-siRNA and percent apoptosis of MCF-7 cells co-cultured with calcarea carbonica-primed T cells in the presence of caspase-9 inhibitor (Z-LEHD-FMK). Values are mean ±SEM of five independent experiments. *p < 0.05 and **p < 0.001 when compared with respective placebo/drug-treated sets.

After establishing the mode of calcarea carbonica-induced cancer cell apoptosis, next we aimed at delineating the underlying mechanism. In previously described co-culture experiments maintaining isogenic conditions, we verified by Western blot analysis the changes in p53 expression in cancer cells alongside with its transcription target Bax, also a major effector of mitochondria-mediated death. Cancer cells with functional p53, upon exposure with calcarea carbonica-primed T cells, demonstrated increase in the expression of p53. Moreover, increase in the levels of p53 transcription target, Bax, was perceived both at protein and mRNA levels (Figure 6A) in functional p53-expressing cells, thereby leading towards the possibility of Bax transactivation by p53 under such conditions. Moreover, these cancer cells upon treatment with calcarea carbonica-primed T cells displayed decrease in Bcl-2 levels both at transcriptional and translational levels (Figure 6A) and thereby decreasing Bcl-2: Bax protein ratio (Figure 6B), thus creating a pro-apoptotic environment. Cancer cells co-cultured with control T cells failed to show any significant change in p53 as well as in Bax (Figure 6A). However, p53-mutated cancer cells, even in the presence of calcarea carbonica-primed T cells failed to induce p53 and thus no changes in the levels of Bax was evident (Figure 6A). Moreover, in p53-silenced (Figure 6C) or p53-mutated (Figure 5B) cancer cells, calcarea carbonica-primed T cells failed to induce apoptosis thereby confirming the involvement of p53 in calcarea carbonica-induced cancer cell apoptosis *via* immunomodulatory circuit.

### Calcarea carbonica-primed T cells induced apoptosis by triggering mitochondrial death cascade in cancer cells

Our attempt to map the down-stream signalling pathways of p53-mediated activity, revealed that in tumor cells co-cultured with calcarea carbonica-primed T cells, Bax migrated from cytosol to mitochondria, accompanied by a significant decrease in cytochrome c level in mitochondria and simultaneous increase in the cytosol (Figure 6D). These results suggested that the mitochondrial translocation of Bax might have led to initiation of the death cascade, with the release of cytochrome c in cancer cells as a result of T cell activation by calcarea carbonica. To determine the level of contamination both mitochondrial and cytosolic fractions isolated from tumor cells were run on the same gel with same exposure time (Figure 6D). Involvement of mitochondrial pathway in p53-mediated apoptosis was further confirmed by measuring DiOC_6_ retention of control and calcarea carbonica-primed T cell co-cultured cancer cells in flow cytometry. Calcarea carbonica-primed T cells produced a significant mitochondrial membrane potential (MTP) loss in cancer cells while pre-treatment of the latter with 25 μM cyclosporine A (CsA) that blocks mitochondrial pore formation, abrogated this effect (Figure 6E).

Mapping of the execution phase of apoptosis demonstrated activation of the critical executioner caspase-3 in EAC and HBL-100 cells (caspase-3-wild-type) and caspase-9 in MCF-7 cells (caspase-3 knockout), as was evident from the substantial decrease in pro-caspase-3/9 and increase in caspase-3/9 at protein levels (Figure 6F) in tumor cells co-cultured with calcarea carbonica-primed T cells for 48 hrs. CsA completely blocked activation of executioner caspases in cancer cells as was manifested by decrease in protein levels of active-caspase-3 and 9 in EAC and MCF-7 cells, respectively. Moreover, significant reduction in apoptosis was also evident after CsA pre-treatment in tumor cells co-cultured with calcarea carbonica-primed T cells (Figure 6G). Interestingly, EAC and HBL-100 cells could significantly overcome calcarea carbonica-insult when transfected with caspase-3-siRNA or treated with the pharmacological inhibitor of caspase-3, Z-DEVD-FMK (Figure 6H), as determined by scoring Annexin-V/7-AAD positivity. Similarly significant decrease in calcarea carbonica-induced apoptosis was observed in MCF-7 cells pre-exposed with caspase-9 inhibitor, Z-LEHD-FMK. All these results together indicate that calcarea carbonica treatment switched over the tumor micro-environment towards apoptosis *via* immuno-restoration, thereby culminating in tumor cell killing.

### Validation of results in patient’s biopsy samples

**Figure 7:**
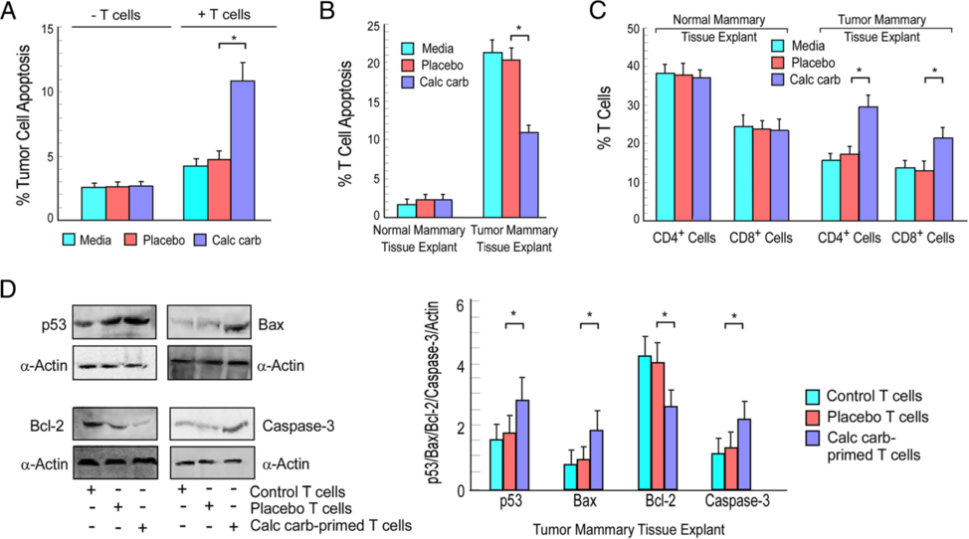
**Calcarea carbonica induces T cell-mediated apoptosis of primary mammary tumor. (A)** T cells isolated from patient’s peripheral circulation were primed with media-/placebo-/calcarea carbonica-treated tumor supernatant for 72 h and then co-cultured with primary mammary tumor for 48 h. In parallel, primary mammary tumor cells was directly exposed to placebo-/calcarea carbonica for 48 h in the absence of T cells. Tumor cell apoptosis was then scored by Annexin-V-PE/7-AAD-positivity and represented graphically. **(B)** T cells isolated from normal and cancer patient’s peripheral blood were co-cultured with control mammary tissue and tumor mammary tissue explants, respectively for 48 h and percent T cell apoptosis was scored by Annexin-V-PE/7-AAD-positivity. **(C)** The same experimental set was analysed for percentage of CD4^+^ and CD8^+^ T cells flow cytometrically. **(D)** Lysates of primary breast cancer cells co-cultured with or without placebo-/calcarea carbonica-primed T cells of same patient’s blood were Western blotted for the analysis of p53, Bax, Bcl-2 and active-caspase-3. α-Actin was used as loading control. *p < 0.05 and **p < 0.001 when compared with respective control/mammary tissue explant sets and placebo/drug-treated sets.

Studies performed in *in vivo or ex-vivo* system were confirmed by reiterating our findings in primary mammary carcinoma and normal mammary tissue samples which were obtained from surgical biopsies with prior consent of patients. The biopsy samples procured were digested to obtain single cells following protocol described in materials and methods section. Each patient’s peripheral blood was also collected to isolate CD3^+^ T cells. Isogenic conditions were maintained throughout co-incubation experiments. Our results demonstrated that T cells primed with calcarea carbonica-treated tumor supernatant induced 12% cell death in carcinoma sample, compared to 4% death by untreated-T cells (Figure 7A). Our previous findings demonstrated that calcarea carbonica failed to exert direct apoptogenic effect. To further validate this in human mammary carcinoma we treated normal and mammary carcinoma cells with calcarea carbonica for 48 h and scored percent apoptosis by Annexin-V/7-AAD assay. Results of Figure 7A re-confirmed that calcarea carbonica induces apoptosis in cancer cells not directly but *via* T cells. We have already shown that calcarea carbonica potentiates depressed immune system of the host and employ it to induce apoptosis in tumor cells. To validate these results next we verified the status of T cell apoptosis when cultured in explants (spent-medium) of control or human mammary carcinoma cells that were untreated or exposed to placebo or calcarea carbonica for 72 h (Figure 7B). In parallel, percentages of CD4^+^ and CD8^+^ T cells were also analyzed (Figure 7C). Results of Figure 7B revealed significant apoptosis in T cells cultured in tumor explants as compared to that in normal tissue explants. Interestingly, calcarea carbonica-exposed tumor explants not only failed to induce T cell apoptosis (Figure 7B) but also increased the percent of CD4^+^ and CD8^+^ T cells (Figure 7C). Our previous findings revealed that calcarea carbonica induced apoptosis in *in vitro* breast cancer cells *via* p53 pathway. To further confirm this in human mammary carcinoma, we co-incubated normal and mammary carcinoma cells with placebo-/calcarea carbonica-primed T cells for 48 h and determined the expressions of p53 and its downstream targets. Our Western blot analysis revealed significant increase in the levels of p53, bax and caspase-3, whereas significant reduction in the levels of bcl-2 was perceived signifying pro-apoptotic environment induced by calcarea carbonica-primed T cells in mammary carcinoma cells when compared to un-/placebo-primed T cells (Figure 7D). Our results therefore support the role of calcarea carbonica in protecting immune cells from tumor insult and to mediate p53-dependent cancer cell apoptosis *via* immumo-modulatory circuit.

## Discussion

Mechanisms that suppress tumorigenesis often involve modulation of signal transduction pathways, leading to alteration in gene expression, arrest in cell cycle progression or apoptosis. There has been extensive research where several homeopathic formulations have been tested for their anti-tumor effects in different cancers describing their direct apoptotic effects on cancer cells [6-10]. Yet another exciting way of amplifying the anti-tumorigenic response is to amplify the immuno-modulatory circuit which as such is severely depressed in tumor conditions but if stimulated can form a strong defense against tumor progression. Tumor progression induces rigorous immunosuppression by inducing apoptosis in T cells [12-14,18,19] and reducing Th1/Tc1 response [20] thereby leading to decrease in activated T cell repertoire [15-17], which includes the abolishment of effective cell-mediated immune response of the host. Cancer-induced immune cell apoptosis as well as block in maturation from CD4^-^8^-^ to CD4^+^8^+^ and finally to CD4^+^ and CD8^+^ effector T cells were also reported [17,20]. Shift of cytokine balance from Th1/Tc1 to Th2/Tc2 has been observed in tumor-bearing mice and in human cancer patients [17,20]. Considering all these information, an approach that has received attention recently provides opportunities to explore the probability of manipulating immune responses of the host against the disease. The prime goal of cancer immunotherapy is to induce apoptosis in tumor cells by recruitment of the host’s immune effector repertoire. However, most of the cancer drugs in use add to such tumor-induced immuno-suppression and concurrently exert toxic manifestations including oxidative stress, liver damage, hepatotoxicity and immunosuppression in the tumor-bearer [36-38]. On the other hand, reports suggest that cancer patients using complementary and alternative medicines (CAM) strengthen immune system [39], alleviate side-effects of chemotherapy, improve quality of life, and help to overcome pain and stress; 62% of them reported subjective beneficial effects [40]. Beside this, immune stimulation by natural products has been attempted in various animal models and in human cancer patients as an adjunct to chemotherapy [41]. Pre-treatment with varying potencies of cadmium has been found to significantly increase lymphocyte viability after toxic challenge compared to control cells [42]. Oliveira *et al.*[43] have recently revealed that highly diluted tinctures can efficiently decrease tumour necrosis factor-alpha (TNF-α) release and IFN-γ production in lipopolysaccharide (LPS)-stimulated macrophages. Application of highly diluted homeopathic medicines to macrophages has been shown to suppress previously elevated levels of TNF-α, increase the activity of NADPH oxidase and the expression of inducible nitric oxide synthase (iNOS), and induce differential gene expression in tumor conditions [11]. Many studies have demonstrated the role of different high diluted complexes in cancer therapy *via* immunomodulation [44-46]. In this context our study for the first time describes how calcarea carbonica by re-arranging the dismantled cell-mediated immune system brings forth active tumor killing. This study, therefore, indicates that calcarea carbonica can be exploited for regression of tumor burden by rejuvenating host’s depressed immune system without inducing systemic toxicity.

Boosting the cell-mediated immune system against tumor cells requires both increasing and activating CTL population. Depleted populations of CD8^+^ cells due to tumor-induced apoptosis have been manifested in different cancers, amelioration of which has revived the anti-tumor potential of CTLs [47]. Consistently we observed that calcarea carbonica ameliorated tumor-induced loss of CD8^+^ T cells. Also the Canova Method, composed of conitum napellus, arsenicum album (arsenic trioxide), bryonia alba, lachesis muta venom and thuja occidentalis, was found to stimulate the depressed immune system of cancer patients by activating macrophages that in turn stimulate lymphocytes for asserting their cytotoxic action against cancer cells [48]. Apart from CD8^+^ cells there is evidence of increased apoptosis among CD4^+^ T cells in peripheral blood lymphocytes from cancer patients and animal models [17]. Concurrently, Bhattacharyya *et al*. [17,18] have reported that tumor derived factors induce helper T cell apoptosis which could be reverted by natural remedies. Because CD4^+^ cells are essential for activation of CTL response, it is noteworthy that calcarea carbonica, in the present study, normalized CD4^+^ cells in different immune organs of tumor-bearing host. Moreover, the immune-boosting efficacy of calcarea carbonica was found to be comparable to the established immune-modulating cytokine, IL2 [17,18]. Reports have also stated that calcarea carbonica and its associations had a promising capacity to stimulate immune cells against melanoma cells both *in vitro* and *in vivo* on melanoma metastasis mouse model [11]. In another study, *in vitro* treatment with calcarea carbonica significantly increased macrophages/lymphocyte interaction and effectiveness against melanoma cells [11].

Other than inducing T cell apoptosis, blocking T cell proliferation is also accountable for reduced T cell populations in tumor patients [49]. Numerous tumor-derived suppressive factors have been reported to prohibit cell signaling responsive for T cell proliferation. Studies showed that tumor condition suppressed expression of JAK3 and tyrosine phosphorylation of STAT5 [50]. Tumor supernatants also partially blocked induction of IL-2R beta and gamma chains expression [50]. Interestingly natural remedies like curcumin and theaflavin were found to restore IL-2 signaling, suggesting that these compounds may inhibit tumor-induced inhibition of T cell proliferation [15-18]. Convincingly we observed that calcarea carbonica restored T cell population by normalizing T cell proliferation. Though T lymphocyte count is a major factor governing anti-tumor responses, it should be remembered that a proper cytokine environment is a must for T cell survival, proliferation and activation. Numerous studies have reported that a type-2 cytokine bias is conducive to tumor growth both by inhibiting production of Th1 cytokines and reduction of CTL function [51]. Calcarea carbonica, however, was found to skew the bias towards type-1 cytokines which implies that inclusion of this drug may necessarily improve the process of tumor immunotherapy.

Tumor sensitivity to cell-mediated immunity often depends on the status of tumor suppressor genes. Importantly the tumor suppressor p53 plays multiple roles in cell cycle control, differentiation, angiogenesis, genomic stability, and apoptosis [31-34]. Mutations of the *p53* gene are frequently found in >50% of all human tumors, suggesting that loss of this gene represents an important step in the formation of human cancers. Thiery *et al*. [28] reported that the restoration of wild-type p53 expression in p53-mutant tumor cells increases tumor susceptibility to CTL-mediated cytolysis. CTL-targeting results in p53 accumulation and activation at very early times. They further showed that p53 is a key determinant in anti-tumor CTL response that regulates induction of Fas receptor expression, cellular FLICE/caspase 8 inhibitory protein short-form degradation, and Bid translocation to target mitochondria [28]. The balance between pro-apoptotic and anti-proliferative genes, activated by p53, is believed to control the choice between apoptosis and growth arrest. It has been shown that p53 triggers apoptosis by inducing mitochondrial outer membrane permeablilization through transcription-dependent and -independent mechanisms [52]. Transcriptional target of p53 include the pro-apoptotic Bcl-2 family member Bax, which translocate to the mitochondria from the cytosol in response to apoptotic signals, permeabilize the outer membrane, resulting in release of mitochondrial proteins such as cytochrome c, AIF etc. in the cytosol or nucleus where they are actively involved in the process of caspase activation and protein/DNA degradation [53]. However, there was still dearth of information regarding whether immuno-modulatory circuit is involved in cancer reverting action of calcarea carbonica and, if any, the underlying molecular mechanisms. We shed light on the molecular mechanism underlying calcarea carbonica-induced immune-therapy of tumor by showing that calcarea-primed T cells executed p53-dependent tumor apoptosis *via* Bax activation and loss of mitochondrial membrane potential that led to augmentation of cytosolic cytochrome c and caspase-3 activation. These results altogether justify the candidature of calcarea carbonica as an anti-cancer agent that induces apoptosis in cancer cells *via* immuno-modulatory circuit.

In the future, experimental as well as clinical studies e.g., using the combination of calcarea carbonica and other homeopathic remedies, will further elucidate its therapeutic value in treating different cancers.

## Conclusion

In summary, our work for the first time indicated an apoptosis-enhancing capability of calcarea carbonica in cancer cells by strengthening the immune system as well as the cross-talk of various pro- and anti-apoptotic factors. Even though further investigations and clinical trials are needed there is an indication that the unique properties of calcarea carbonica can be exploited for regression of tumor burden by rejuvenating host’s depressed immune system without inducing systemic toxicity.

## Abbreviations

Bax: Bcl-2 associated X protein; Bcl-2: B cell lymphoma-2; CAM: Complementary and alternative medicine; CTL: Cytotoxic T lymphocyte; CFSE: Carbonicaoxyfluorescein succinimidyl ester; CsA: cyclosporine A; DAPI: 4’,6-diamidino-2-phenylindole dihydrochloride; DiOC6: 3,3 dihexyloxacarbonicaocyanine iodide; EAC: Ehrlich’s ascites carcinoma; IFN-γ: Interferon gamma; IL-2: Interleukin-2; IL-4: Interleukin-4; MTP: Mitochondrial membrane potential; PBL: Peripheral blood lymphocytes; PCR: Polymerase chain reaction; siRNA: Short-interfering RNA; shRNA: Small hairpin RNA; TCR: T cells expressing antigen specific receptor; Th1: T helper cell type-1; Th2: T helper cell type-2; TNF: Tumor necrosis factor.

## Competing interests

The authors declare that they have no competing interests.

## Authors’ contribution

SS conceived the study, performed *in vivo* experiments, cell culture studies, Western blot experiments, analyzed the data and drafted the manuscript. DMSH performed *in vivo* experiment and participated in flow cytometric studies during immuno-phenotyping and analyzed the data. SM participated in cell culture and Western blot experiments. SM participated in cell culture experiments and analyzed the results. MM, SM and UKG assisted in *in vivo* experiments. CR, CN, AK and RC analysed the results and reviewed the manuscript. GS and TD conceived the study, supervised the experiments, analyzed the data and helped to draft the manuscript. All authors have read and approved the manuscript.

## Pre-publication history

The pre-publication history for this paper can be accessed here:

http://www.biomedcentral.com/1472-6882/13/230/prepub
